# Label-free fiber-optic spherical tip biosensor to enable picomolar-level detection of CD44 protein

**DOI:** 10.1038/s41598-021-99099-x

**Published:** 2021-10-01

**Authors:** Aliya Bekmurzayeva, Zhannat Ashikbayeva, Zhuldyz Myrkhiyeva, Aigerim Nugmanova, Madina Shaimerdenova, Takhmina Ayupova, Daniele Tosi

**Affiliations:** 1 School of Engineering and Digital Sciences, Nazarbayev University, Nur-Sultan, 010000 Kazakhstan; 2 National Laboratory Astana, Nazarbayev University, Nur-Sultan, 010000 Kazakhstan

**Keywords:** Biomedical engineering, Sensors and probes

## Abstract

Increased level of CD44 protein in serum is observed in several cancers and is associated with tumor burden and metastasis. Current clinically used detection methods of this protein are time-consuming and use labeled reagents for analysis. Therefore exploring new label-free and fast methods for its quantification including its detection in situ is of importance. This study reports the first optical fiber biosensor for CD44 protein detection, based on a spherical fiber optic tip device. The sensor is easily fabricated from an inexpensive material (single-mode fiber widely used in telecommunication) in a fast and robust manner through a CO_2_ laser splicer. The fabricated sensor responded to refractive index change with a sensitivity of 95.76 dB/RIU. The spherical tip was further functionalized with anti-CD44 antibodies to develop a biosensor and each step of functionalization was verified by an atomic force microscope. The biosensor detected a target of interest with an achieved limit of detection of 17 pM with only minor signal change to two control proteins. Most importantly, concentrations tested in this work are very broad and are within the clinically relevant concentration range. Moreover, the configuration of the proposed biosensor allows its potential incorporation into an in situ system for quantitative detection of this biomarker in a clinical setting.

## Introduction

Biomarker detection is becoming one of the crucial clinical assessment tools in all aspects of clinical medicine, such as disease prevention, diagnosis, and treatment^1^. Despite the progress in cancer therapy, tumor progression and metastasis remain the leading causes of cancer death. Advancements made in genomics, transcriptomics, proteomics, and metabolomics have facilitated the discovery of new biomarkers^2^, including those in cancer. Mounting evidence showed that a cluster of differentiation 44 (CD44) molecule is an important tumor protein associated with metastasis. CD44 is a family of transmembrane glycoproteins, which is encoded by the *CD44 gene* on human chromosome 11. CD44 has several ligands, including fibronectin, osteopontin, chondroitin, and hyaluronic acid (HA)^3^. CD44 is expressed in many types of human cells including embryonic stem cells and cancer cells. Accumulating evidence has shown that abnormal expression of CD44 contributes to cancer progression and metastasis, including ovarian cancer, breast cancer, lung adenocarcinoma, glioblastoma, and colorectal cancer. It has been supported by many studies that CD44 is an important biomarker of cancer stem cells (CSC) and is particularly important in CSC microenvironment communication. CSC plays a crucial role in cancer progression and relapse because of its ability to self-renew and differentiate into multiple types of tumor cells. Moreover, CD44’s pivotal role in epithelial-mesenchymal transition has a significant impact on tumor progression^4^. Up to the present time, different sensors and platforms were developed to detect CD44-expressing cells. This includes quartz crystal microbalance-based sensor^5^ and magnetic-fluorescent iron oxide–carbon hybrid nanoparticles^6^ and colorimetric nanobiosensor^7^ for the detection of CD44^+^ breast cancer cells, functionalized interdigitated electrodes for prostate cancer cells expressing CD44^8^, functionalized stainless steel wire to capture breast CSC^9^ among others.

In addition to the expression of CD44 protein on the cell surface, soluble CD44 protein is also present in serum. It was suggested that shedding rather than internalization is an important regulatory mechanism responsible for the downregulation of this cell surface adhesion molecule^10^. Shedding of CD44 is mediated by membrane-associated metalloproteases and is observed in many types of cancer^11^. The soluble form of CD44 protein in serum is examined by a conventional enzyme-linked immunosorbent assay (ELISA) using antibodies for quantitative analysis. Detecting CD44 in serum was proposed as a simple, non-invasive way to study tumor burden and metastasis in gastric and colon cancer^12^. Elevated expression of CD44 in serum has been shown to associate with the presence of distant metastases and tumor reoccurrence^13^. Serum CD44 in patients with advanced colon or gastric cancer showed considerably increased concentration levels when compared to healthy people (30.8 nM and 24.4 nM respectively versus 2.7 nM). Surgical resection of the tumor in patients with gastric or colon cancer resulted in a significant decrease of CD44 concentration in serum. Moreover, the concentration level of soluble CD44 in patients with cirrhosis was demonstrated to be 2.2 nM^12^. Many studies reported that colon cancer progression and metastasis are influenced by the amount of CD44 protein expression^14^. A study of CD44 in breast cancer patients reported a direct correlation between serum CD44 level and breast cancer occurrence^15^. Investigation of breast cancer patients with the metastatic disease showed a considerably increased level of serum CD44. Liver and bone metastases in patients with breast cancer were associated with increased concentration of CD44 in serum^16^. Furthermore, an increased CD44 protein level in serum was observed in patients with non-Hodgkin’s lymphoma^17^ and cervical cancer^18^.

Tremendous work is done in the field of cancer diagnostics and treatment; however, there is an emerging need for an accurate biomarker detection method. The development of new methods to detect cancer biomarkers could potentially improve cancer screening, diagnosis, and treatment^19^. In order to be clinically significant, a biomarker detection tool must have high predictive accuracy and be minimally invasive and easily measurable. Conventional biomarker detection technology for the detection of CD44 protein levels in serum is based on ELISA. ELISA inherits such limitations as having worse performance in terms of limit of detection (LoD), and obtained results can be affected by non-specific interactions^20^, and it requires series of complex operations and is able to measure only one analyte at a time^21^, therefore developing technologies with new capabilities that overcome ELISA will provide better opportunities to detect CD44 in serum. A biosensor can provide a real-time monitor of a condition with an excellent capacity for detection^19^. Developing biosensors for CD44 protein detection has attracted attention only in recent years. Their performance indicates that they can be a good alternative to ELISA reaching a far lower LoD than ELISA. Zhang et al. developed an electrochemical sensor with multi-walled carbon nanotubes assembled on the indium tin oxide (TiO_2_) electrode surface using HA to detect CD44 in serum, thus conjugating the carbon nanotubes with HA-CD44 ligand–protein interaction^22^. Another sensor was proposed by^23^ when a photoelectrochemical antifouling surface based on the HA and poly(ethylene glycol) were immobilized on TiO_2_ substrate. In another study, Soomro et al.^24^ used an *insitu* approach to form hybrid photoactive material for their photoelectrochemical biosensor for CD44 detection. Zhou et al.^25^, on the other hand, developed an electrochemical biosensor using immobilized aptamers for label-free detection of CD44.

Among different biosensing platforms, an optical fiber biosensor is a good alternative for an easy and cheap diagnostic, as it does not necessitate electrical connections and is not affected by electric interference like electrochemical biosensors^26^. The inherent advantages of optical fiber biosensors include biocompatibility, small size, compactness, lightness, resistance to electromagnetic interference, low cost of production. Moreover, optical fiber biosensors can detect more than one analyte at a time by building a multiplexed assay^27,28^. Optical fibers allow in situ and real-time sensing through a suitable packaging in a medical device, as shown for example by Loyez et al^29^ who designed an endoscopic device embedding a fiber optic biosensor, or by Liao et al.^30^ who designed a subcutaneous package for a fiber optic fluorosensor through a hydrogel. In addition, optical fiber biosensors report excellent performance rates^31^ in terms of LoD, detection speed, and selectivity. The main optical fiber platforms used in the biosensor applications include surface plasmon resonance (SPR), interferometers, and different grating-based optical fiber sensors such as etched fiber Bragg gratings (FBG), tilted FBG (TFBG), long-period grating (LPG), and plasmonic FBG^32–34^.

Fiber optic biosensors have been exploited as sensor platforms for a variety of applications such as sensing glucose^35–42^, sucrose^36^, heavy metal ions such as lead, copper, cobalt, cadmium ^43–45^, amino acids ^46^, cholesterol^47^, triacylglycerides^48^, urea^49^, ascorbic acid^50^, DNA^34,51^, proteins including thrombin^52–54^, cancer biomarkers^55,56^ and cytokeratin^57,58^, antibodies^59^, bacterial^60–62^ and mammalian cells^63,64^.

While each of the system offers its own advantages, they also have some limitations. All grating-based sensors require inscription to produce periodic modulation of the refractive index within the core of an optical fiber using specialized equipment^65^ adding up to its manufacturing cost. Tilted FBG and LPG work in transmission making it less practical in terms of serving as a sensing probe. An additional step of fabricating a broadband mirror on the tip of the cleaved fiber is required for TFBG and LPG to work in reflection^66^. A precise cut of LPG after the grating to avoid the formation of interference fringes is required before coating it with a reflecting layer^67^. Tapers and SPR sensors, while being the main alternatives to grating-based sensors, are harder to manufacture, and tapers are fragile and have a low fabrication yield^68^.

In this work, we present the detection of CD44 protein using anti-CD44 antibody on a fiber optic spherical tip. This sensor works in reflection thus allowing its use in vivo. Also, it does not necessitate the inscription of gratings inside the fiber core as in grating-based optical fiber sensors. Most importantly, the platform is fabricated in a fast and easy way requiring only a telecommunication-grade fiber and splicing machine. This is done by aligning and splicing two fibers, further heating the produced structure with the high-power laser while undergoing breaking close to the splicing point to produce a fiber optic spherical tip. This sensor is sensitive to refractive index (RI) change of the surrounding media making it suitable as a biosensing platform after functionalization. An experimental setup used for biosensor development is based on the interrogation of this sensor with optical backscatter reflectometry (OBR) for measuring signal during calibration and protein measurements. Surface modification steps used to develop the biosensor included surface pre-treatment (silanization and gold coating), crosslinker binding, immobilization of antibodies against CD44, and blocking. To the best of our knowledge, this is the first study on the detection of CD44 based on an optical fiber biosensor.

## Results

### Fiber-optic spherical tip design and profile

**Figure 1:**
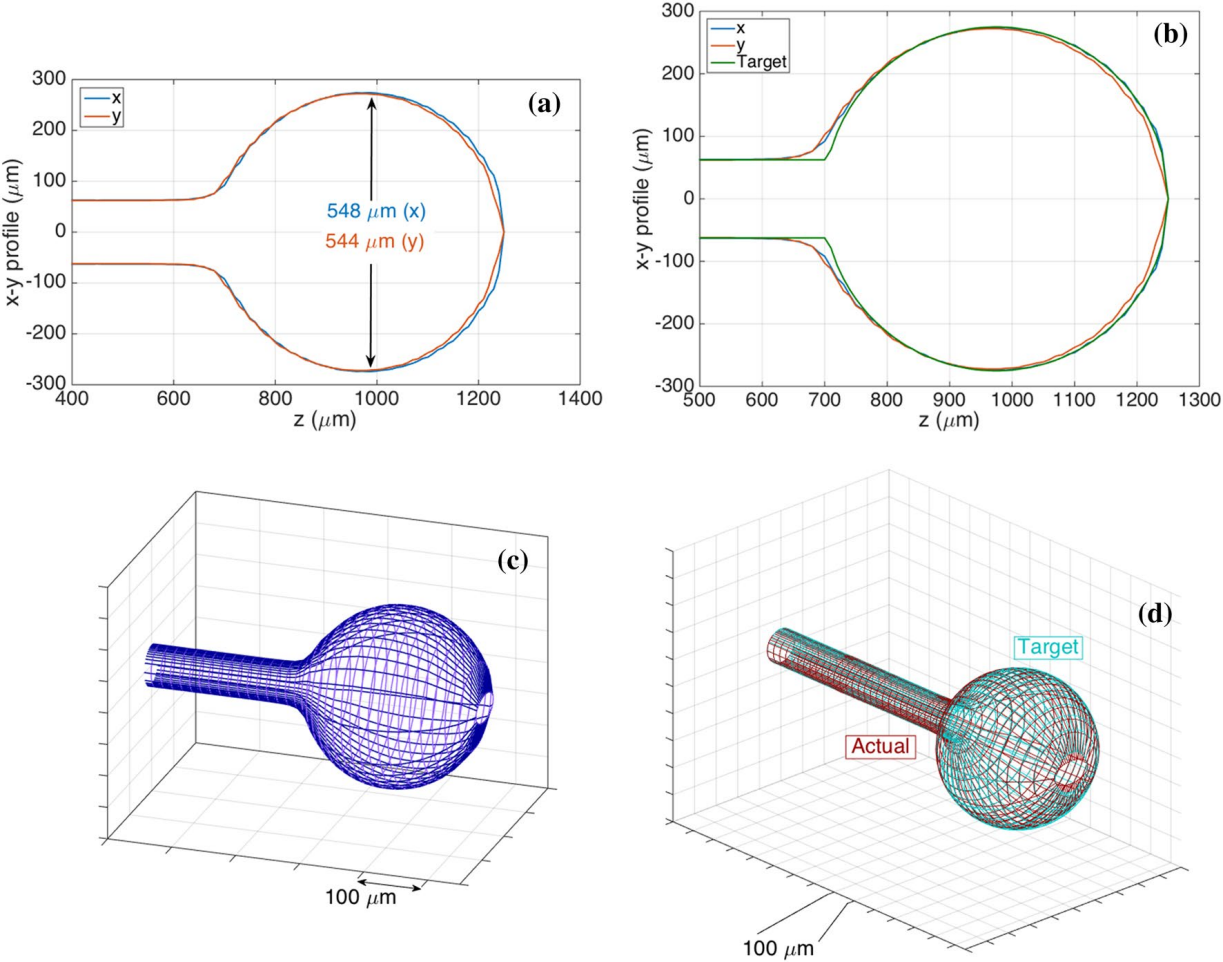
The geometrical profile of fiber optic spherical tip sensor (548–544 µm) used to detect CD44 protein in this work. **(a,b)** Two-sided profilometry of the fiber optic tips obtained from Fujikura splicer as measured by its inner microscope; where diameter on the horizontal and vertical axes (x, y) for each position along the fiber axis (z) is shown; **(a)** actual sensor (fabricated and used in experiments) and **(b)** actual sensor vs. target (i.e. designed profile for the splicing fabrication). **(c,d)** 3D profiles extrapolated from profilometry data by reconstructing the elliptical meshes of the tips: **(c)** actual sensor and **(d)** actual sensor vs. target; Ellipticity548-544 µm = 0.1206.

In this work, the possibility to develop an optical fiber biosensor for the detection of CD44 protein was investigated. For this, a sphere was fabricated on the tip of single-mode fiber (SMF) as was demonstrated in previous work ^32^ which acted as a weak interferometer. We used this platform as a transducer element to build a biosensor. For this, we used a commercial splicing system for fabrication and optical backscatter reflectometry for interrogation. Two-sided profilometry and 3D profiles (extrapolated from profilometry data) of the spherical tips of the main sensor (used for CD44 protein measurement) fabricated in this work are shown in Fig. 1. Figure S1 shows geometrical profiles of control sensors (used for measurement of control proteins) which were further functionalized in a similar manner as the main sensor**.**

### Calibration of a fiber-optic spherical tip

**Figure 2:**
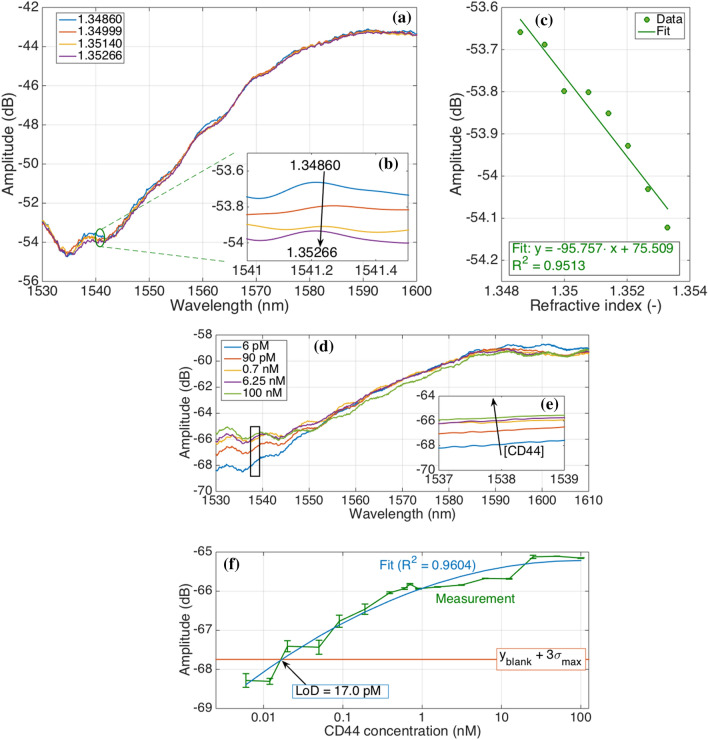
Performance of fiber optic spherical tip sensor in terms of RI sensitivity and CD44 protein detection. **(a–c)** RI calibration of fiber optic spherical tip (548–544 µm) after gold coating in different sucrose concentrations: 10.49% to 13.53% in 8 steps of 0.49%, corresponding to RI values of 1.34860 to 1.35329, for a total change of 4.69 × 10^–3^ RIU in steps of 5.86 × 10^–4^ RIU. **(a)** Spectra showing the change of amplitude of the sensor in four sucrose concentrations; **(b)** inset showing integrated spectral response in the range where the sensor had the highest response (between 1541 and 1541.5 nm) for sensitivity estimation; **(c)** amplitude change as a function of RI change; curve processed with linear regression, R2 = 0.9513 with an estimated sensitivity = 95.76 dB/RIU. **(d–f)** CD44 protein detection by fully functionalized spherical fiber optic tip biosensor; **(d)** Amplitude change occurring during measurement of different concentrations of CD44 protein by the biosensor; **(e)** an inset showing integrated spectral response in the range where the sensor had the most sensitive response (between 1537 and 1539 nm) for LoD estimation; **(f)** amplitude change as a function of protein concentration; the blue line represents the fitting of the experimental data by using second-order polynomial equation.

After silanization and gold-coating, the tip was calibrated in different sucrose solutions having different RI values (Fig. 2a–c). With the increased RI of the solutions, the amplitude of the signal is lowered. Control sensors were also studied and Figure S2 shows RI calibration results for these sensors.

### Surface morphology and FITC analysis

**Figure 3:**
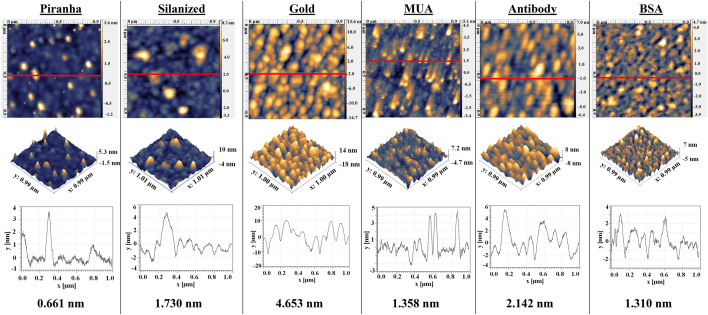
AFM micrographs of spherical optical fiber tips at each step of functionalization; Upper row: 1 µm × 1 µm images; second row: their 3D images; third row: height variation across the red line; and bottom row: root mean square roughness. *MUA* 11-mercaptoundecanoic acid, *BSA* bovine serum albumin.

Silanization of the surface was analyzed by FITC analysis where Piranha-treated (control surface) and APTMS-treated tips were incubated with FITC; results are shown in Figure S3. Each functionalization step of the spherical tip was also studied by AFM and results can be seen in Fig. 3. Silanized surface shows an increased roughness compared to fiber after Piranha treatment. After silanization, a thin layer of gold was sputtered on the tip and surface roughness further increased with particles evenly covering the surface with sizes ranging from 10 to 20 nm. After MUA treatment the surface becomes smoother. After antibody immobilization, particles with a height of 4–6 nm were observed on the surface, and after blocking the surface becomes smoother.

### CD44 protein detection

Fully functionalized optical fiber spherical tips were used for the detection of the target molecule (CD44 protein by the main sensor) as well as two control proteins (thrombin and IL-4 by control sensors). Spectral change occurring during measurement of different CD44 protein concentrations by the fully functionalized sensor is shown in Fig. 2d–f. A rise in amplitude as the protein concentration increases can be seen. Spectral response in the range where the sensor had the most sensitive response (between 1537 and 1539 nm) was integrated to determine the LoD of the sensor which was calculated to be 17 pM (Fig. 2f). At low concentration (below 0.1 nM), the sensitivity of 1.23 dB for each 10 × increase of concentration was observed. Results of analyzing amplitude change for the middle protein concentration (0.8 nM) as a function of time were studied in more detail and demonstrated a very small fluctuation of the signal (0.12 dB) for the whole time of measurement (Figure S4). Whole spectra during different steps of fabrication were also analyzed and results are shown in Figure S5.

### Specificity studies

**Figure 4:**
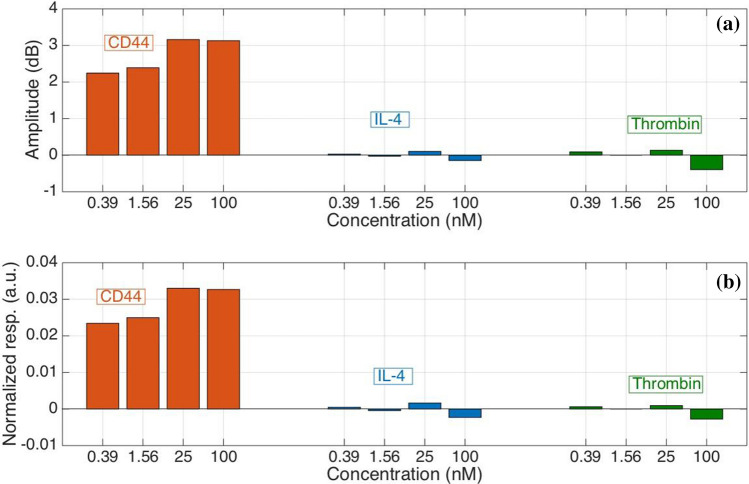
Studying specificity of functionalized fiber optic tip biosensor for CD44 protein detection by measuring control proteins (IL-4 and thrombin). The reference protein concentration used was 6 pM. (**a**) Amplitude change of the sensors when measuring target protein vs. control proteins in different concentrations; (**b**) Response of the sensors normalized to their respective RI sensitivities.

Spectral changes of the control sensors functionalized in the same way as the main sensor are shown in Figure S6. In contrast to the main sensor, control sensors do not have a distinct amplitude change associated with the increased protein concentration. Figure 4 shows a comparison of the integrated spectral responses of the main sensor vs. control sensors. In contrast to the main sensor, these proteins do not induce a significant amplitude change as can be seen from Fig. 4a. The response to these proteins is fluctuating: ranging from negative to positive values; while the main sensor has a homogenous response where reflectivity increases with the increased protein concentration resulting in the specificity of the biosensor to be 4.9%. All error bars in this chart have amplitude within ± 0.1 dB.

Since all sensors had different sensitivities to RI (shown in Fig. 2a–c and Figure S2), the effect of sensitivities on the resulting signal change with the increased protein concentration was studied. Figure 4b demonstrates the response for three proteins normalized by the RI sensitivity of each sensor respectively. The results ultimately show that the performance of the fabricated biosensor is specific towards the analyte of interest rather than control proteins irrespective of the initial RI sensitivities of the sensors.

## Discussion

The current work investigated the possibility of the optical fiber biosensor development for the detection of CD44 protein. Using a splicing machine to fabricate such sensors offers advantages over the fabrication of grating-based optical fiber sensors because of reduced fabrication time; it is also more favorable to interferometers because of their higher fabrication yield^32^. To make the tip of the fabricated sensor act as a weak interferometer and make it sensitive to RI change, its surface was coated with a thin layer of gold. It was demonstrated that gold nanoparticles have low adhesion to optical fibers and the use of silane-coupling agents (SCA) can be a good choice as a linker^69^. For an improved gold adhesion on the optical fiber surface, such SCA as (3-mercaptopropyl) trimethoxysilane (MPTMS)^70–72^, APTMS^70,73^, and 3-aminopropyltriethoxysilane (APTES)^74^ were used in different studies. In this work, we used APTMS which has a hydrolysable alkoxy group at one end and an amine group at the other end to improve gold adhesion on optical fiber. After gold-coating, sensors were calibrated in solutions with different RI values and the integrated spectral response was used for estimation of the sensor’s sensitivity which was calculated to be 95.76 dB/RIU (Fig. 2c). The estimated sensitivity of the main sensor is in between the sensitivity values of the two control sensors. Together, these results suggested the applicability of these sensors for further use as a biosensing platform once functionalized, and show a superior sensitivity figure with respect to tilted fiber Bragg gratings^58^ and U-bent fiber probes^75^.

The next step in building a biosensor after demonstrating sensitivity to RI change was the functionalization of optical fiber with ligand to specifically bind the analyte of interest. Several receptor immobilization methods on fiber-optic biosensors were utilized in the past: adsorption, electrostatic self-assembly through an ionic bond, cross-linking by multifunctional reagent, covalent attachment, and biotin-avidin linkage^76^. Surface modification of gold-coated optical fiber includes two main strategies: physical adsorption and covalent attachment. Covalent attachment can be done in a one-step or two-step approaches. The two-step approach includes the use of an intermediate layer, bifunctional molecules (linkers) able to react both with the gold and the bioreceptor^66^. One of the most common linkers is MUA which has a thiol group to attach on gold and carboxyl groups to further bind the ligand. Carboxyl groups on MUA can further be activated by incubating the surface with EDC and NHS before incubation with antibodies as was shown in different studies^38,77,78^. AFM is very useful in determining nanoscale changes on the modified surfaces^66^. It was used to study the change of morphology in terms of surface roughness and height of the attached particles after each step of surface treatment and demonstrated a fully functionalized surface (Fig. 3). Silanization of the surface was also demonstrated by FITC analysis (Figure S3). FITC is a fluorescent dye with N = C = S functional group which reacts with amine groups (such as those present on APTMS). Therefore, after silanization and incubation with FITC, the surface can be further visualized using a fluorescence microscope. Similarly, FITC analysis was done after silanization using for qualitative analysis of various surfaces including such surfaces as silicon oxide^79^, poly(dimethylsiloxane)^80^, nanoparticles^81^, and titanium^82^.

Numerous studies suggest an important role of CD44 protein in serum as a good biomarker of tumor burden and metastasis^83^. Also, the main method of serum protein detection in many studies was ELISA using commercial kits (from Abnova Corporation^84,85^; Bender MedSystems^86,87^) which measures standard CD44 and all its isoforms. Quantitation of CD44 by ELISA requires sample preparation (serum dilution, incubation, washing) and takes more than 3 h. Having both enzymes and antibodies also increases its cost and complexity^23^. Using other analytical tools such as biosensors which offer rapid detection, portability, and an on-site test could be a good alternative to ELISA. Existing biosensors to detect this protein include at least four biosensors based on differential pulse voltammetry (DPV), electrochemical impedance spectroscopy (EIS), photoelectrochemical sensor (PEC); their functionalization and performance are shown in Table 1. All existing biosensors are works done very recently. The main strengths of these biosensors lie in their high sensitivities, being tested in such complex environments as serum. In one of the studies, an electrochemical signal amplified with carbon nanotubes was constructed and tested on both soluble proteins and CD44 expressing cells^22^. Another biosensor combined HA with antifouling properties of poly(ethylene glycol) to build a hybrid surface with excellent performance^23^.

**Table 1:** Currently available biosensors developed for detection of CD44 protein. Listed by the date of publication (from older to more recent).

Method/sensor type	Sensor surface	Sensor size	Functionalization used (ligand in bold)	LoD	Concentration range	References
DPV & EIS	ITO	7.5 mm × 25 mm	MWCNT-PDDA-**HA**-BSA	5.94 pg/mL	0.01 − 100 ng/mL	^22^
PEC & EIS	ITO	2.5 cm × 0.8 cm	TiO_2_ NP-PDA-**HA**-PEG	0.44 pg/mL	0.005–500 ng/mL	^23^
EIS	Gold	Diameter: 3 mm	**Aptamers (**thiolated)	87 pg/mL	0.1–1000 ng mL	^25^
PEC	ITO	0.5 cm × 0.5 cm Thickness: 1.5 mm	TiO_2_/MX-BiVO_4_-**HA**	1.4 × 10^−2^ pg/mL	2.2 × 10^−4^ to 3.2 ng/mL	^24^
Fiber-optic spherical tip	OF	Fiber 125 µm Tip diameter: ~ 550 µm	APTMS-gold-MUA-**AbCD44**-BSA	17 pM (390 pg/mL)	0.006–100 nM (138 pg/mL to 2300 ng/mL)	Current work

*AbCD44* antibodies against CD44 protein, *APTMS* (3-aminopropyl)triethoxysilane, *DPV* differential pulse voltammetry, *EIS* electrochemical impedance spectroscopy, *HA* hyaluronic acid, *ITO* indium tin oxide, *MUA* mercaptoundecanoic acid, *MWCNT* multiwalled carbon nanotubes, *NP* nanoparticles, *OF* optical fiber, *PDA* polydopamine, *PDDA* poly(diallyldimethylammonium chloride, *PEC* photoelectrochemical.

Biosensor based on spherical fiber optic tip fabricated in this work demonstrated stable response during the whole protein measurement with a very small fluctuation of the signal (Figure S4). Whole spectra during different steps of fabrication (Figure S5) show that after gold sputtering (before functionalization), the spectrum in the air is higher (1.2 dB) than that for water. This must be due to a low RI of air. After the sensor is functionalized its reflectivity is lowered (difference 15.7 dB) probably due to an additional layer of molecules used during functionalization. LoD achieved by the functionalized spherical fiber optic tip was 17 pM. Although LoD is much higher than that of the other CD44 biosensors, it is still enough to detect the lowest clinically relevant CD44 level in the reported studies. Earlier studies showed serum CD44 levels in normal individuals is 2.7 nM versus 24.2 nM in advanced gastric and 30.8 colon cancer^12^. Other studies showed the median serum protein levels in healthy people being as high as 178 ng/ml^88^, 260 ng/mL^89^, 275 ng/mL^87^, or 437.9 ng/mL^90^. This discrepancy in the results might be due to different factors including ELISA kits with different performance, choosing criteria for normal individuals, number of chosen samples, the difference in CD44 isoforms present in serum, etc. Soluble CD44 found in 140 breast cancer patients ranged from 220.8 ng/mL to 1216.7 ng/mL while the median serum level was ≥ 417.4 ng/mL with different levels of the protein in different subtypes of breast cancer (406.4 ng/mL in luminal, 506.8 ng/mL in triple-negative and 462.5 ng/mL in HER2-enriched subtype)^85^. In patients with B-cell chronic lymphocytic leukemia, the median CD44 level in serum was 450 ng/mL^86^. The median serum level of CD44 in non-Hodgkin lymphomas was 540 ng/mL^91^. The concentration range used in the current work is much broader than the available CD44 biosensors and most importantly concentration range covers the clinically relevant concentration of this protein. The performance figures reported in this work meet these requirements for CD44 detection, as the sensor operates in a relatively wide operation range that encompasses the low concentrations; the spectrum appears to slightly saturate for concentrations higher than 50–100 nM, which might be a common effect in some of the high-sensitivity fiber optic biosensors such as those reported by Lobry et al.^40,56^.

Although EIS, DPV, and PEC offer very high sensitivity in terms of protein detection, optical fiber sensors seems a more advantageous platform for application in real clinical application. Electroactive neurochemicals can be determined in vivo analysis by electrochemical sensors/biosensors (DPV, EIS) but this is mostly done in neurological fluids/tissues^92^. Due to electroactive interference problems, ascorbic acid, uric acid, and some drugs present in the blood can cause problems to electrochemical sensors ^93^. Optical fiber sensors, on the other hand, have this potential because they are electrically safe and their small size allows them to be used in vivo where electric current is detrimental^26,94^. Using optical fiber as a biosensing platform also offers such advantages as low cost, chemical and electromagnetic inertness and a variety of applicable surface modification methods, and the potential to be used for remote sensing^95,96^. Moreover, optical fibers can be miniaturized and multiplexed to detect several targets simultaneously^27,28^. Moreover, the sensing region of this sensor is located on the tip; and having a sensing region of the optical fiber at one end makes it suitable for use towards in situ and in clinical applications^97^. Optical fiber-based biosensors for potential in situ applications were demonstrated including miniaturized systems for antibody measurement^98^ and thrombin sensing^53^, bronchoscope-embedded sensor^29^, percutaneous glucose sensing^30^.

The performance of the biosensor was tested using two control proteins which are not the main targets of the anti-CD44 antibody. Specificity studies are vital in order to validate the efficiency of the fabricated biosensor to specifically bind the analyte of interest. The conducted trials allowed to conclude that the surface of the biosensor functionalized with anti-CD44 antibody demonstrated high binding performance to its target of interest—CD44 protein compared to the control proteins. Furthermore, the performance levels of the three sensors were not due to the difference in the inherent sensitivities to RI (Fig. 4b) but were due to the specificity of the ligand-analyte system.

## Methods

### Fabrication of fiber optic spherical tip

Fiber optic spherical tip biosensors were fabricated at the end-point of the standard single-mode fibers (SMF-28) using a CO_2_ laser splicer (Fujikura LZM-100). This was done by aligning two fibers, splicing, and then subjecting the produced structure to high laser power to form a spherical tip at the end of the single-mode fiber when it underwent breaking close to the splicing point. High laser power is specific to the fabrication equipment. The chosen parameters are shown in Table S1, presenting the values of absolute and relative powers, speed of rotation, and feeding speed to obtain the spherical tip sensors of diameters 548–544 µm, 490–484 µm, and 525–520 µm. Absolute power was determined by power calibration before the fabrication procedure that was 342 bit for all the fibers. The optical fiber with the diameter 544–548 µm was employed further for the CD44 protein detection, while fibers with the diameters 490–484 µm and 525–520 µm were utilized for the control measurements of interleukin-4 (IL-4) protein and thrombin protein measurements respectively. The fabrication process of spherical tip sensors has a short duration (~ 60 s), as it is derived from well-known routines for ball lens fabrications adapted for smaller single-mode fibers.

### Interrogation using optical backscattering reflectometer and data analysis

**Figure 5:**
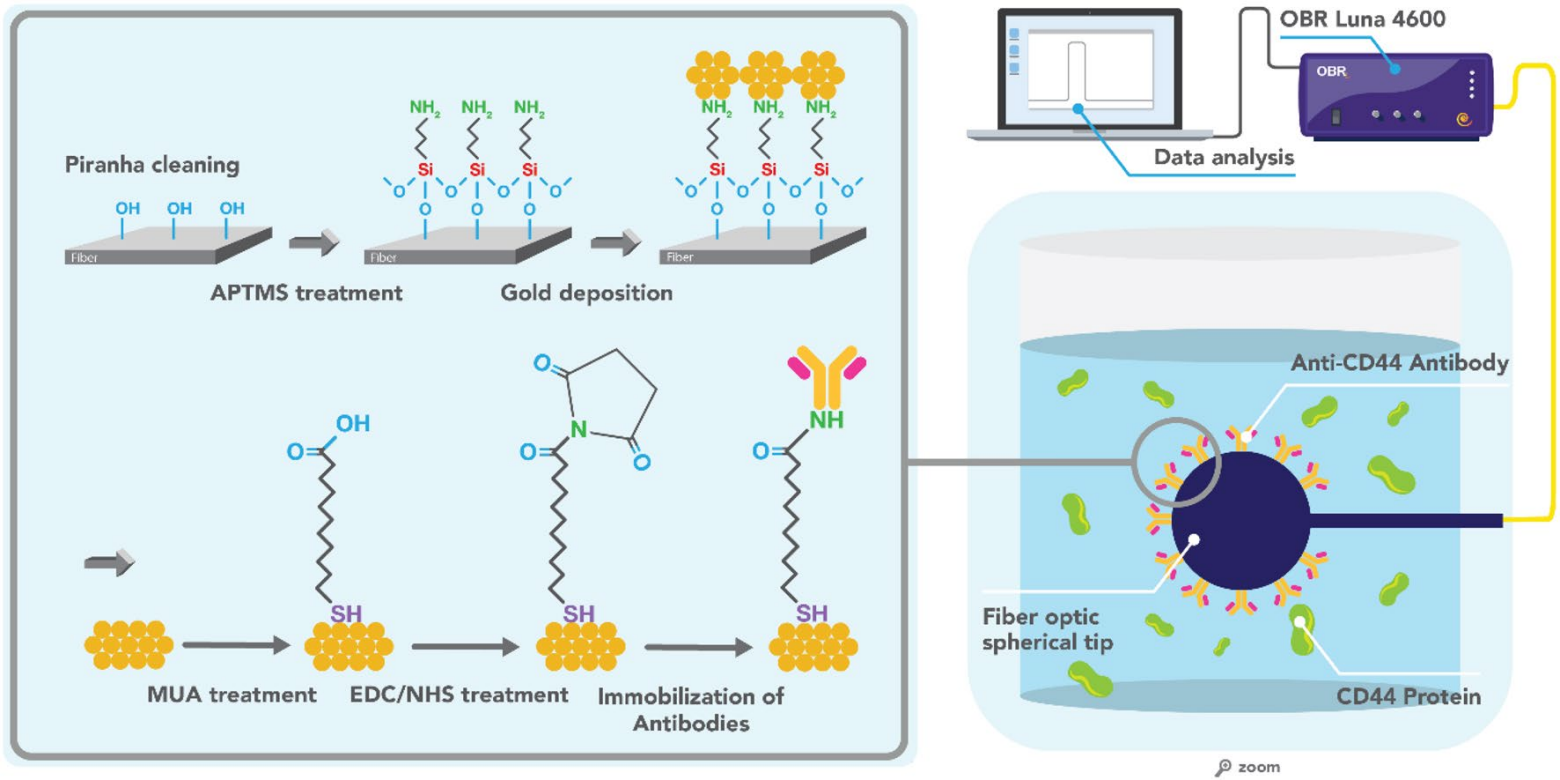
The experimental setup used for the CD44 protein measurements using a fiber optic spherical tip-based biosensor. Surface functionalization steps are shown in the zoomed area. *APTMS* 3-(aminopropyl)trimethoxysilane, *MUA 11-mercaptoundecanoic acid*, *EDC *1-ethyl-3-(3-dimethylaminopropyl)carbodiimide hydrochloride, *NHS* *N*-hydroxysuccinimide, *OBR* optical backscatter reflectometry.

Optical backscatter reflectometer (OBR) (LUNA OBR 4600) was used for interrogation of the system during RI calibration and protein measurements (Fig. 5). The following OBR parameters were used: scan range 1525–1610 nm, 0 dB gain, and resolution bandwidth 0.258 GHz; in total 65,536 data points were collected. Polarization P (parallel, with respect to the Luna laser) spectra were chosen for analysis. Data have been processed with a low-pass filter (Chebyshev type 1, 7th order, with 0.0084 digital frequency cut-off. Spectral features have been identified using a feature tracking method that highlights the most significant spectral feature.

### Pre-treatment and gold deposition

Optical fibers with the fabricated spherical tip at the end underwent surface cleaning using freshly prepared Piranha solution (must be handled with extreme caution) to remove the organic residuals and increase surface hydroxyl groups. Optical fibers attached to the glass rods were placed into the beaker for 15 min containing 30 ml of Piranha solution (H_2_SO_4_:H_2_O_2_ = 4:1) and followed by a thorough cleaning with deionized (DI) water. The cleaned and dried with nitrogen gas optical fiber spherical tips were treated with (3-aminopropyl)trimethoxysilane (APTMS) (1% in methanol) for 20 min aimed to introduce an amine group on the surface of the tip before gold deposition. Afterward, the optical fibers were cleaned with methanol, and heat-treated for 40 min at 110 °C, followed by the DI water rinsing. Finally, the fiber optic spherical tips were coated with gold at 30 nm thickness using the sputtering machine (Q150T Plus, Quorum Technologies Ltd). The gold-coated optical fibers were further annealed at 200 °C for 2 h in the oven to uniformly distribute the gold layer. The surface pre-treatment of fiber optical spherical tip is presented in Fig. 5.

### Sensor calibration

After gold coating, the sensors were calibrated by measuring the change of spectra to refractive index change (RI) using 6.1 ml of 10.49% sucrose solution followed by the stepwise addition of 100 µl of 40% sucrose solution in the manually fabricated vial. Overall, sucrose solutions starting from 10.49% to 13.53% in 8 increments of 0.49% were tested that corresponded to RI values of 1.34860 to 1.35329, for a total change of 4.69 × 10^–3^ RIU in steps of 5.86 × 10^–4^. For control sensors, calibration was done for 5 RI values (1.34860 to 1.35140 in 5 data points). Integrated spectral responses in the range where sensors had the highest responses (between 1541 and 1541.5 nm for the main sensor; 1560–1652 nm for thrombin sensor and 1549–1550 nm for IL-4 sensor) were used for sensitivity estimation. In addition, the measurement of RI change using fiber optic spherical tip was performed in DI water and PBS solution.

### Surface functionalization of the fiber optic spherical tip with CD44 antibodies

Before the antibody immobilization onto the surface of the fiber optic sensing tip, the pre-treated optical fibers were placed into 11-mercaptoundecanoic acid (MUA) solution (9.2 mM in ethanol) for 16 h at 2–4 °C to achieve effective covalent binding followed by the activation with 1-ethyl-3-(3-dimethylaminopropyl)carbodiimide hydrochloride (EDC) (250 mM) and *N*-hydroxysuccinimide (NHS) (100 mM) for 15 min. Finally, the fiber optic spherical sensing tip was incubated on a shaker with 8 mg/ml anti-CD44 antibody for 30 min, unreacted sites were blocked with 1% bovine serum albumin (BSA), washed, and stored in phosphate buffer saline (PBS) at 2–4 °C. All fibers were functionalized in the same way before different protein measurements.

### Studying surface morphology

Surface morphology change of the fiber-optic spherical tip after each step of surface functionalization was observed using SmartSPM 1000 (AIST-NT Inc., Novato, CA, USA) scanning probe microscope. Super sharp high-resolution cantilever “NSG30_SS” (TipsNano company) was used as a probe in alternating current mode. The scanning parameters used were as follows: scanning rate 1 Hz, scan area 1 μm × 1 μm in the X–Y plane.

### Fluorescein-5-isothiocyanate (FITC) analysis after silanization

Optical fiber after silanization with APTMS was incubated with FITC (125 µg/mL; Sigma Aldrich, Steinheim, Germany) in sodium carbonate/bicarbonate buffer (pH 9.2) for 2 h in the dark and washed with ethanol for 5 min according to^9^. Piranha-treated fiber (no silanization) served as a control sample. Samples were visualized using a fluorescence microscope (Leica DM4000 B Digital Microscope).

### CD44 detection using functionalized fiber optic spherical tip

Functionalized fiber optic spherical tips were used to measure target protein (544–548 µm sensor) or two control proteins (thrombin by 525–520 µm and IL-4 by 490–484 µm sensors) respectively. The spectral processing was conducted in the 1537–1539 nm range. The CD44 protein at concentrations from 0.006 nM to 100 nM in PBS in 4-times (4×) dilution was placed into a manually fabricated 200 µl vial to perform the measurements. The measurements were taken after 10 min for each concentration in triplicates. The 2nd order polynomial fit was done using an equation: y = f(x) = ax^2^ + bx + c, where x is the log10 of CD44 concentration in nM, from 6 pM to 100 nM over 4 orders of magnitudes, and y is the spectral response of the sensor in dB units. The fitted values of the parameters is: a = −0.1786, b = 0.7118, c = −65.927 (R^2^ = 0.9604).

LoD was estimated using a method proposed by Chiavaioli et al.^31^ that was applied to the fit: LoD = f^−1^(y_blank_ + 3σ_max_); where y_blank_ is the response at the lowest CD44 concentration (6 pM used as a reference) where the sensor is almost unresponsive and σ_max_ is the maximum of the standard deviation recorded in the experiments = 0.538 dB.

### Specificity studies using control proteins

IL-4 and thrombin were used as control proteins to validate the specificity of the fiber optical spherical tip biosensor. For this, two control sensors underwent the same functionalization treatment as the main sensor, and amplitude changes occurring when measuring three proteins (0.39; 1.56; 25 and 100 nM) by the three sensors were compared. Spectral responses in the range between 1558 and 1573 nm and 1560–1575 nm (for IL-4 and thrombin respectively) were integrated to compare the amplitude change between three sensors. The amplitude change was measured from the reference value, which was taken as 6 pm for all sensors. During the study, the response to each concentration for IL-4 and thrombin proteins was reported comparing with the CD44 protein concentrations.

Response of the sensors was normalized to their respective RI sensitivities to compare biosensors’ performance irrespective of their inherited sensitivities. Both the bare response (in dB units), and the normalized response were reported where each sensor response is divided by its sensitivity, to assess comparatively the response to controls against the response to CD44 protein.

The specificity was estimated by comparing the normalized response of the sensor at 25 nM (where all sensors record a positive intensity change) of the highest control (0.00163 a.u.) to the normalized response of the CD44 protein at the same concentration (0.03301 a.u.).

## Conclusion

The current study, to the best of our knowledge, is the first biosensor based on an optical fiber sensor for the detection of CD44 protein. The fiber-optic spherical tip is used as a sensing platform in this work. Its fabrication can be done in a fast and robust way and requires only a CO_2_ laser splicing machine and telecommunication-grade fibers. After showing its sensitivity to RI change, the sensor was functionalized with specific antibodies. Application of optical fiber sensing tip for the detection of CD44 in different concentrations showed an increase in spectral amplitude with increasing concentrations of the analyte. Furthermore, the concentrations of two control proteins were measured with the optical fiber biosensor, resulting in no substantial change in the obtained signal. The proposed convenient and cost-effective optical fiber biosensor offers a novel promising way in the detection of an important biomarker. The developed CD44 biosensor was able to detect a clinically relevant range of the protein. With further optimization of the performance, a fiber-optic spherical tip biosensor could be used for monitoring the levels of biomarkers in situ, and in real-time by implementing the spectral tracking on board of the OBR instrument.

## Supplementary Information

Supplementary Information.

## References

[1] Chen X-H, Huang S, Kerr D (2011). Biomarkers in clinical applications. IARC Sci. Publ..

[2] Wang K, Huang CH, Nice EC (2014). Proteomics, genomics and transcriptomics: Their emerging roles in the discovery and validation of colorectal cancer biomarkers. Expert Rev. Proteomics.

[3] Xu HX, Niu MK, Yuan X, Wu KM, Liu AG (2020). CD44 as a tumor biomarker and therapeutic target. Exp. Hematol. Oncol..

[4] Yan YM, Zuo XS, Wei DY (2015). Concise review: Emerging role of CD44 in cancer stem cells: A promising biomarker and therapeutic target. Stem Cells Transl. Med..

[5] Yang XJ, Zhou RC, Hao Y, Yang PH (2017). A CD44-biosensor for evaluating metastatic potential of breast cancer cells based on quartz crystal microbalance. Sci. Bull..

[6] Han CP (2019). Multifunctional iron oxide-carbon hybrid nanoparticles for targeted fluorescent/MR dual-modal imaging and detection of breast cancer cells. Anal. Chim. Acta.

[7] Rauta PR, Hallur PM, Chaubey A (2018). Gold nanoparticle-based rapid detection and isolation of cells using ligand-receptor chemistry. Sci. Rep..

[8] Neto J (2020). Polysaccharide multilayer films in sensors for detecting prostate tumor cells based on hyaluronan-CD44 interactions. Cells.

[9] Bekmurzayeva A (2020). Optimizing silanization to functionalize stainless steel wire: Towards breast cancer stem cell isolation. Materials..

[10] Bazil V, Horejsi V (1992). Shedding of the CD44 adhesion molecule from leukocytes induced by anti-CD44 monoclonal antibody simulating the effect of a natural receptor ligand. J. Immunol..

[11] Stamenkovic I, Yu Q (2009). Shedding light on proteolytic cleavage of CD44: The responsible sheddase and functional significance of shedding. J. Investig. Dermatol..

[12] Guo Y (1994). Potential use of soluble CD44 in serum as indicator of tumor burden and metastasis in patients with gastric or colon cancer. Can. Res..

[13] Ristamaki R, Joensuu H, Jalkanen S (1999). Serum CD44 in non-Hodgkin's lymphoma. Leuk. Lymphoma.

[14] Matsumura Y, Tarin D (1992). Significance of CD44 gene products for cancer diagnosis and disease evaluation. Lancet.

[15] Mayer S (2008). Increased soluble CD44 concentrations are associated with larger tumor size and lymph node metastasis in breast cancer patients. J. Cancer Res. Clin. Oncol..

[16] Lackner C (1998). Soluble CD44 v5 and v6 in serum of patients with breast cancer. Correlation with expression of CD44 v5 and v6 variants in primary tumors and location of distant metastasis. Breast Cancer Res. Treat..

[17] Ristamaki R, Joensuu H, Lappalainen K, Teerenhovi L, Jalkanen S (1997). Elevated serum CD44 level is associated with unfavorable outcome in non-Hodgkin's lymphoma. Blood.

[18] Kainz C (1995). Serum CD44 splice variants in cervical cancer patients. Cancer Lett..

[19] Goossens N, Nakagawa S, Sun XC, Hoshida Y (2015). Cancer biomarker discovery and validation. Translat. Cancer Res..

[20] Nimse SB, Sonawane MD, Song KS, Kim T (2016). Biomarker detection technologies and future directions. Analyst.

[21] Leng SX (2008). ELISA and multiplex technologies for cytokine measurement in inflammation and aging research. J. Gerontol. Ser. A Biol. Sci. Med. Sci..

[22] Zhang R (2019). Label-free electrochemical sensor for CD44 by ligand-protein interaction. Anal. Chem..

[23] Fan BB (2019). Photoelectrochemical biosensor for sensitive detection of soluble CD44 based on the facile construction of a poly(ethylene glycol)/hyaluronic acid hybrid antifouling interface. ACS Appl. Mater. Interfaces..

[24] Soomro RA (2020). In-situ engineered MXene-TiO2/BiVO4 hybrid as an efficient photoelectrochemical platform for sensitive detection of soluble CD44 proteins. Biosens. Bioelectron..

[25] Zhou J (2020). Determination of soluble CD44 in serum by using a label-free aptamer based electrochemical impedance biosensor. Analyst.

[26] Mowbray SE, Amiri AM (2019). A brief overview of medical fiber optic biosensors and techniques in the modification for enhanced sensing ability. Diagnostics..

[27] Marazuela MD, Moreno-Bondi MC (2002). Fiber-optic biosensors - An overview. Anal. Bioanal. Chem..

[28] Mehrvar M, Bis C, Scharer JM, Moo-Young M, Luong JH (2000). Fiber-optic biosensors - Trends and advances. Anal. Sci..

[29] Loyez M (2019). In situ cancer diagnosis through online plasmonics. Biosens. Bioelectron..

[30] Liao K-C (2008). Percutaneous fiber-optic sensor for chronic glucose monitoring in vivo. Biosens. Bioelectron..

[31] Chiavaioli F, Gouveia CAJ, Jorge PAS, Baldini F (2017). Towards a uniform metrological assessment of grating-based optical fiber sensors: From refractometers to biosensors. Biosensors-Basel..

[32] Shaimerdenova M, Ayupova T, Sypabekova M, Tosi D (2020). Fiber optic refractive index sensors based on a ball resonator and optical backscatter interrogation. Sensors..

[33] Chiavaioli F, Baldini F, Tombelli S, Trono C, Giannetti A (2017). Biosensing with optical fiber gratings. Nanophotonics.

[34] Chen, X. *et al.* EDC-mediated oligonucleotide immobilization on a long period grafting optical biosensor. *J. Biosens. Bioelectron.* **6** (2015).

[35] Luo BB, Yan ZJ, Sun ZY, Li JF, Zhang L (2014). Novel glucose sensor based on enzyme immobilized 81 degrees tilted fiber grating. Opt. Express.

[36] Badmos AA (2017). Enzyme-functionalized thin-cladding long-period fiber grating in transition mode at dispersion turning point for sugar-level and glucose detection. J. Biomed. Opt..

[37] Chen KC, Li YL, Wu CW, Chiang CC (2018). Glucose sensor using U-shaped optical fiber probe with gold nanoparticles and glucose oxidase. Sensors..

[38] Khan MRR, Watekar AV, Kang SW (2018). Fiber-optic biosensor to detect pH and glucose. IEEE Sens. J..

[39] Cao SQ (2018). Highly sensitive surface plasmon resonance biosensor based on a low-index polymer optical fiber. Opt. Express.

[40] Lobry M (2019). Non-enzymatic D-glucose plasmonic optical fiber grating biosensor. Biosens. Bioelectron..

[41] Pahurkar VG, Tamgadge YS, Gambhire AB, Muley GG (2015). Glucose oxidase immobilized PANI cladding modified fiber optic intrinsic biosensor for detection of glucose. Sens. Actuat. B-Chem..

[42] Zhang XJ (2018). Hydrogen peroxide and glucose concentration measurement using optical fiber grating sensors with corrodible plasmonic nanocoatings. Biomed. Opt. Express.

[43] Kuswandi B, Andres R, Narayanaswamy R (2001). Optical fibre biosensors based on immobilised enzymes. Analyst.

[44] Tan RX, Ibsen M, Tjin SC (2019). Optical fiber refractometer based metal ion sensors. Chemosensors..

[45] Benounis M, Jaffrezic-Renault N, Halouani H, Lamartine R, Dumazet-Bonnamour I (2006). Detection of heavy metals by an optical fiber sensor with a sensitive cladding including a new chromogenic calix 4 arene molecule. Mater. Sci. Eng. C-Biomimet. Supramol. Syst..

[46] Sharma P, Semwal V, Gupta BD (2019). A highly selective LSPR biosensor for the detection of taurine realized on optical fiber substrate and gold nanoparticles. Optic. Fiber Technol..

[47] Semwal V, Gupta BD (2018). LSPR- and SPR-based fiber-optic cholesterol sensor using immobilization of cholesterol oxidase over silver nanoparticles coated graphene oxide nanosheets. IEEE Sens. J..

[48] Baliyan A, Sital S, Tiwari U, Gupta R, Sharma EK (2016). Long period fiber grating based sensor for the detection of triacylglycerides. Biosens. Bioelectron..

[49] Botewad SN, Pahurkar VG, Muley GG (2018). Fabrication and evaluation of evanescent wave absorption based polyaniline-cladding modified fiber optic urea biosensor. Opt. Fiber Technol..

[50] Zhu G (2020). A novel periodically tapered structure-based gold nanoparticles and graphene oxide—Immobilized optical fiber sensor to detect ascorbic acid. Opt. Laser Technol..

[51] Bertucci A (2015). Detection of unamplified genomic DNA by a PNA-based microstructured optical fiber (MOF) Bragg-grating optofluidic system. Biosens. Bioelectron..

[52] Sypabekova M (2019). Functionalized etched tilted fiber Bragg grating aptasensor for label-free protein detection. Biosens. Bioelectron..

[53] Shevchenko Y (2011). In situ biosensing with a surface plasmon resonance fiber grating aptasensor. Anal. Chem..

[54] Lao, J. J. *et al.* Gold nanoparticle-functionalized surface plasmon resonance optical fiber biosensor: In situ detection of thrombin with 1 nM detection limit. *J. Lightwave Technol.* **37**, 2748–2755. 10.1109/jlt.2018.2822827 (2019).

[55] Sridevi S, Vasu KS, Asokan S, Sood AK (2015). Sensitive detection of C-reactive protein using optical fiber Bragg gratings. Biosens. Bioelectron..

[56] Lobry M (2020). Multimodal plasmonic optical fiber grating aptasensor. Opt. Express.

[57] Loyez M, Albert J, Caucheteur C, Wattiez R (2018). Cytokeratins biosensing using tilted fiber gratings. Biosensors-Basel..

[58] Caucheteur C, Loyez M, Gonzalez-Vila A, Wattiez R (2018). Evaluation of gold layer configuration for plasmonic fiber grating biosensors. Opt. Express.

[59] Liu LL (2018). Highly sensitive label-free antibody detection using a long period fibre grating sensor. Sens. Actuators B-Chem..

[60] Balasubramanian S, Sorokulova IB, Vodyanoy VJ, Simonian AL (2007). Lytic phage as a specific and selective probe for detection of *Staphylococcus aureus—*A surface plasmon resonance spectroscopic study. Biosens. Bioelectron..

[61] Tripathi SM (2012). Long period grating based biosensor for the detection of *Escherichia coli* bacteria. Biosens. Bioelectron..

[62] Ahmed A, Rushworth JV, Hirst NA, Millner PA (2014). Biosensors for whole-cell bacterial detection. Clin. Microbiol. Rev..

[63] Loyez M (2020). Rapid detection of circulating breast cancer cells using a multiresonant optical fiber aptasensor with plasmonic amplification. Acs Sens..

[64] Malachovska V (2015). Fiber-optic SPR immunosensors tailored to target epithelial cells through membrane receptors. Anal. Chem..

[65] Chen, X. in *Current Developments in Optical Fiber Technology* (eds. Harun, W., & Arof, H.) (2012).

[66] Albert J, Lepinay S, Caucheteur C, DeRosa MC (2013). High resolution grating-assisted surface plasmon resonance fiber optic aptasensor. Methods.

[67] Quero G (2016). Long period fiber grating working in reflection mode as valuable biosensing platform for the detection of drug resistant bacteria. Sens. Actuators B-Chem..

[68] Bekmurzayeva A (2018). Etched fiber Bragg grating biosensor functionalized with aptamers for detection of thrombin. Sensors.

[69] Bonyar, A., Wimmer, B. & Csarnovics, I. Development of a localised surface plasmon resonance sensor based on gold nanoparticles. in *Proceedings of the 2014 37th International Spring Seminar on Electronics Technology (ISSE)—Advances in Electronic System Integration*. 371–376 (2014).

[70] Cao J, Tu MH, Sun T, Grattan KTV (2013). Wavelength-based localized surface plasmon resonance optical fiber biosensor. Sensors Actuators B Chem..

[71] Cao J, Zhao D, Qin YY (2019). Novel strategy for fabrication of sensing layer on thiol-functionalized fiber-optic tapers and their application as SERS probes. Talanta.

[72] Luo BB (2018). A novel immunosensor based on excessively tilted fiber grating coated with gold nanospheres improves the detection limit of Newcastle disease virus. Biosens. Bioelectron..

[73] Lepinay S, Staff A, Ianoul A, Albert J (2014). Improved detection limits of protein optical fiber biosensors coated with gold nanoparticles. Biosens. Bioelectron..

[74] Houngkamhang N (2018). Gold-nanoparticle-based fiber optic sensor for sensing the refractive index of environmental solutions. Chiang Mai J. Sci..

[75] Zhang C (2017). U-bent fiber optic SPR sensor based on graphene/AgNPs. Sens. Actuators B Chem..

[76] Maguis S (2008). Biofunctionalized tilted fiber Bragg gratings for label-free immunosensing. Opt. Express.

[77] Han LZ (2017). Specific detection of aquaporin-2 using plasmonic tilted fiber grating sensors. J. Lightwave Technol..

[78] Tyagi D (2018). Nano-functionalized long-period fiber grating probe for disease-specific protein detection. J. Mater. Chem. B.

[79] Baumgartel T, von Borczyskowski C, Graaf H (2013). Selective surface modification of lithographic silicon oxide nanostructures by organofunctional silanes. Beilstein J. Nanotechnol..

[80] Seguin C, McLachlan JM, Norton PR, Lagugne-Labarthet F (2010). Surface modification of poly(dimethylsiloxane) for microfluidic assay applications. Appl. Surf. Sci..

[81] Xu WJ (2012). Amine surface modifications and fluorescent labeling of thermally stabilized mesoporous silicon nanoparticles. J. Phys. Chem. C.

[82] Heller M (2018). Osseous response on linear and cyclic RGD-peptides immobilized on titanium surfaces in vitro and in vivo. J. Biomed. Mater. Res. Part A.

[83] Senbanjo LT, Chellaiah MA (2017). CD44: A multifunctional cell surface adhesion receptor is a regulator of progression and metastasis of cancer cells. Front. Cell Dev. Biol..

[84] Baek JM, Jin QR, Ensor J, Boulbes DR, Esteva FJ (2011). Serum CD44 levels and overall survival in patients with HER2-positive breast cancer. Breast Cancer Res. Treat..

[85] Kong YA (2018). Breast cancer stem cell markers CD44 and ALDH1A1 in serum: distribution and prognostic value in patients with primary breast cancer. J. Cancer.

[86] Eisterer W (2004). Elevated levels of soluble CD44 are associated with advanced disease and in vitro proliferation of neoplastic lymphocytes in B-cell chronic lymphocytic leukaemia. Leuk. Res..

[87] Dasari S, Rajendra W, Valluru L (2014). Evaluation of soluble CD44 protein marker to distinguish the premalignant and malignant carcinoma cases in cervical cancer patients. Med. Oncol..

[88] Sawant S (2018). Prognostic significance of elevated serum CD44 levels in patients with oral squamous cell carcinoma. J. Oral Pathol. Med..

[89] Masson D (1999). Soluble CD44: Quantification and molecular repartition in plasma of patients with colorectal cancer. Br. J. Cancer.

[90] Molica S, Vitelli G, Levato D, Giannarelli D, Gandolfo GM (2001). Elevated serum levels of soluble CD44 can identify a subgroup of patients with early B-cell chronic lymphocytic leukemia who are at high risk of disease progression. Cancer.

[91] Shah N (2012). Prognostic value of serum CD44, intercellular adhesion molecule-1 and vascular cell adhesion molecule-1 levels in patients with indolent non-Hodgkin lymphomas. Leuk. Lymphoma.

[92] Xiao TF (2017). In vivo analysis with electrochemical sensors and biosensors. Anal. Chem..

[93] Yin MJ, Huang BB, Gao SR, Zhang AP, Ye XS (2016). Optical fiber LPG biosensor integrated microfluidic chip for ultrasensitive glucose detection. Biomed. Opt. Express.

[94] Biran, I., Yu, X. & Walt, D. *Optical Biosensors Today and Tomorrow* (eds. Ligler, F. & Taitt, C.) Chap. 1. (Elsevier, 2008).

[95] Chryssis AN (2005). Detecting hybridization of DNA by highly sensitive evanescent field etched core fiber Bragg grating sensors. IEEE J. Sel. Top. Quantum Electron..

[96] Leung A, Shankar PM, Mutharasan R (2007). A review of fiber-optic biosensors. Sens. Actuators B. Chem..

[97] Zhang YJ, Hsu JC, Tsao JH, Sun YS (2019). Fabrication of a bare optical fiber-based biosensor. Micromachines..

[98] Zeni L (2020). A portable optical-fibre-based surface plasmon resonance biosensor for the detection of therapeutic antibodies in human serum. Sci. Rep..

