# Publisher Correction: Label-free fiber-optic spherical tip biosensor to enable picomolar-level detection of CD44 protein

**DOI:** 10.1038/s41598-021-00914-2

**Published:** 2021-11-11

**Authors:** Aliya Bekmurzayeva, Zhannat Ashikbayeva, Zhuldyz Myrkhiyeva, Aigerim Nugmanova, Madina Shaimerdenova, Takhmina Ayupova, Daniele Tosi

**Affiliations:** 1 School of Engineering and Digital Sciences, Nazarbayev University, Nur-Sultan, 010000 Kazakhstan; 2 National Laboratory Astana, Nazarbayev University, Nur-Sultan, 010000 Kazakhstan

Correction to: *Scientific Reports* 10.1038/s41598-021-99099-x, published online 1 October 2021

The original version of this Article contained an error in the legend of Figure 4 which was a duplication of the legend of Figure 5.

“The experimental setup used for the CD44 protein measurements using a fiber optic spherical tip-based biosensor. Surface functionalization steps are shown in the zoomed area. *APTMS* 3-(aminopropyl)trimethoxysilane, *MUA 11-mercaptoundecanoic acid*, *EDC* 1-ethyl-3-(3-dimethylaminopropyl)carbodiimide hydrochloride, *NHS* *N*-hydroxysuccinimide, *OBR* optical backscatter reflectometry.”

now reads:

“Studying specificity of functionalized fiber optic tip biosensor for CD44 protein detection by measuring control proteins (IL-4 and thrombin). The reference protein concentration used was 6 pM. (a) Amplitude change of the sensors when measuring target protein *vs.* control proteins in different concentrations; (b) Response of the sensors normalized to their respective RI sensitivities.”

The original Article has been corrected.

