# Supplementary material for: Label-free fiber-optic spherical tip biosensor to enable picomolar-level detection of CD44 protein
Source: Sci Rep. 2021 Oct 1;11:19583. doi: 10.1038/s41598-021-99099-x (PMC8486867; doi:10.1038/s41598-021-99099-x)
Supplement: Supplementary file 1 — Supplementary Information. [file 41598_2021_99099_MOESM1_ESM.pdf]

Label-free fiber-optic spherical tip biosensor to enable picomolar-level detection of CD44 protein

Aliya Bekmurzayeva[a,#,*], Zhannat Ashikbayeva[a,#,*], Zhuldyz Myrkhiyeva[a], Aigerim Nugmanova[a], Madina Shaimerdenova[a], Takhmina Ayupova[a], Daniele Tosi[a,b.]

[a] School of Engineering and Digital Sciences, Nazarbayev University, Nur-Sultan, Kazakhstan;

[b] National Laboratory Astana, Nazarbayev University, Nur-Sultan, Kazakhstan;

[#] Equally contributed to the work;

[*] Corresponding authors; emails: abekmurzayeva@nu.edu.kz; zhashikbayeva@nu.edu.kz

Table S1. Parameters used to fabricate fiber optic spherical tips using CO_2 laser splicer (Fujikura LZM-100).

**Parameters**	**Diameters (*x*, *y* axes), µm**		
	**490-484**	**525-520**	**548-544**
Pre-heat (bit)	1	1	1
Absolute power (bit)	342	342	342
Relative power (bit)	80	60	153
Break-add power (bit)	80	10	130
Feeding speed (mm/sec)	0.2	0.1	0.2
Rotator speed (deg./sec)	90	65	150
Diameter adjustment (µm)	10	10	10

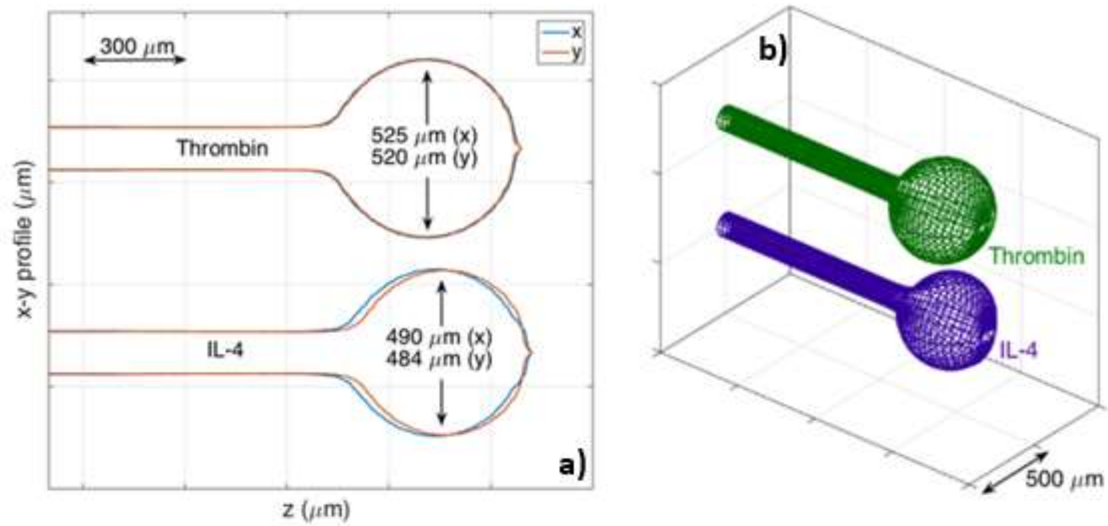

Figure S1. Geometrical profile of control fiber optic spherical tip sensors (525-520 µm and 490-484 µm) functionalized with CD44 antibodies used to detect control proteins (thrombin and IL-4 respectively). (a) Two-sided profilometry of the fiber optic tips obtained from Fujikura splicer as measured by its inner microscope; where diameter on the horizontal and vertical axes (*x*, *y*) for each position along the fiber axis (*z*) is shown; (b) 3D profiles extrapolated from profilometry data by reconstructing the elliptical meshes of the tips for two control sensors; $\text{Ellipticity}_{525\text{-}520\ \mu m} = 0.1376$; $\text{Ellipticity}_{490\text{-}484\ \mu m} = 0.1560$

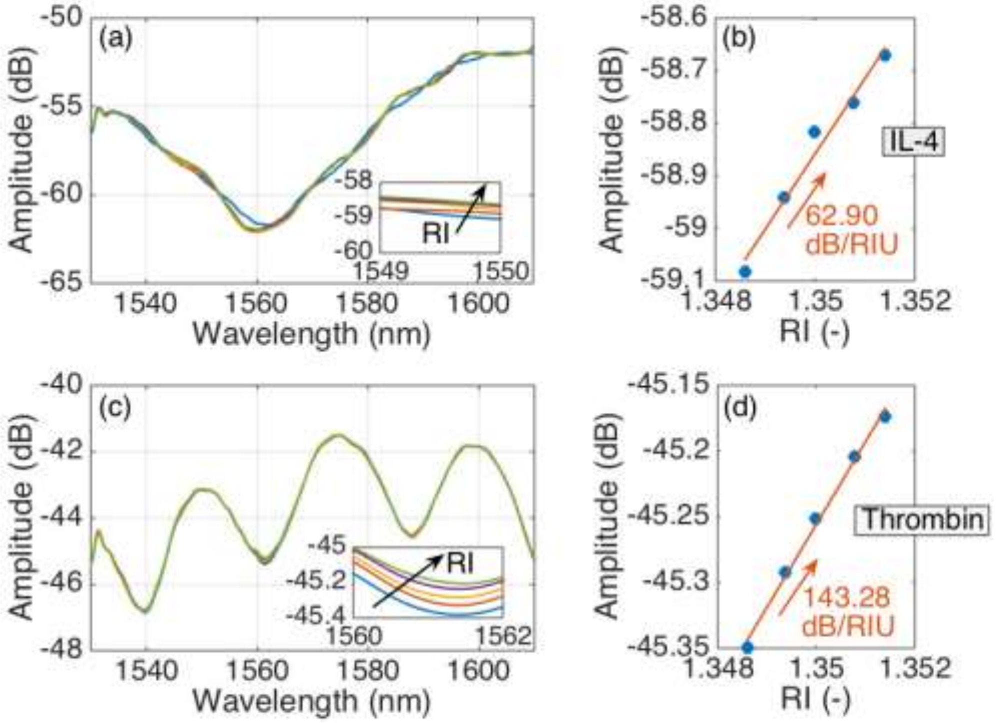

Figure S2. RI calibration of control fiber optic spherical tip sensors (**a** and **b**: 525-520 µm further functionalized for thrombin measurement; **c** and **d:** 490-484 µm further functionalized for IL-4 measurement). Different sucrose concentrations were used starting from 10.49% in 5 steps corresponding to RI values of 1.34860 to 1.35140; (**a, c**) Amplitude spectra of the sensors in different sucrose concentrations; Inset showing integrated spectral response in the range where sensors had the highest response for sensitivity estimation; (**b**, **d**) Amplitude change as a function of RI change; curves processed with linear regression; for thrombin, R^2 = 0.9733 with an estimated sensitivity of 143.918 dB/RIU while for IL-4, R^2 = 0.9513 with an estimated sensitivity of 62.90 dB/RIU. Arrows show the direction of the RI trend.

Piranha + FITC **Piranha + APTMS + FITC**

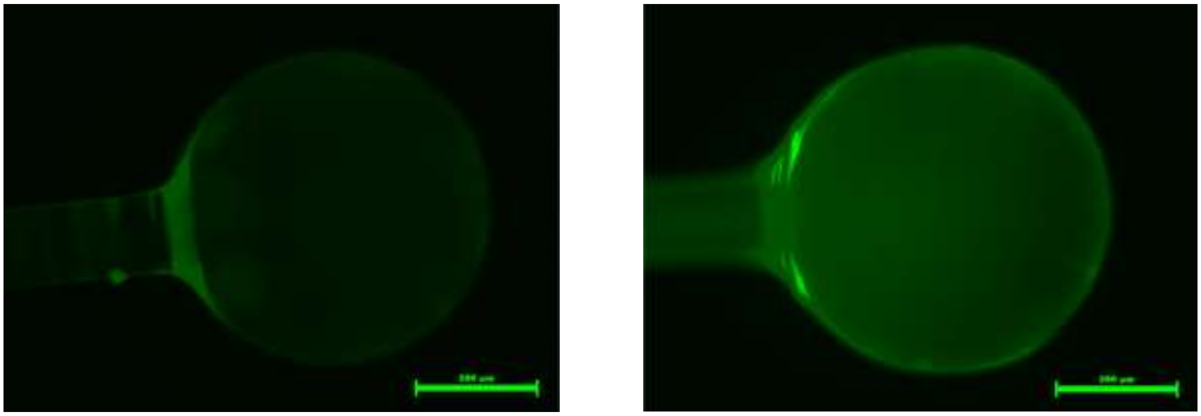

Figure S3. FITC analysis of the control (after Piranha treatment) and silanized fiber optic tips (Piranha + APTMS) as visualized by fluorescence microscopy.

APTMS - (3-Aminopropyl)trimethoxysilane.

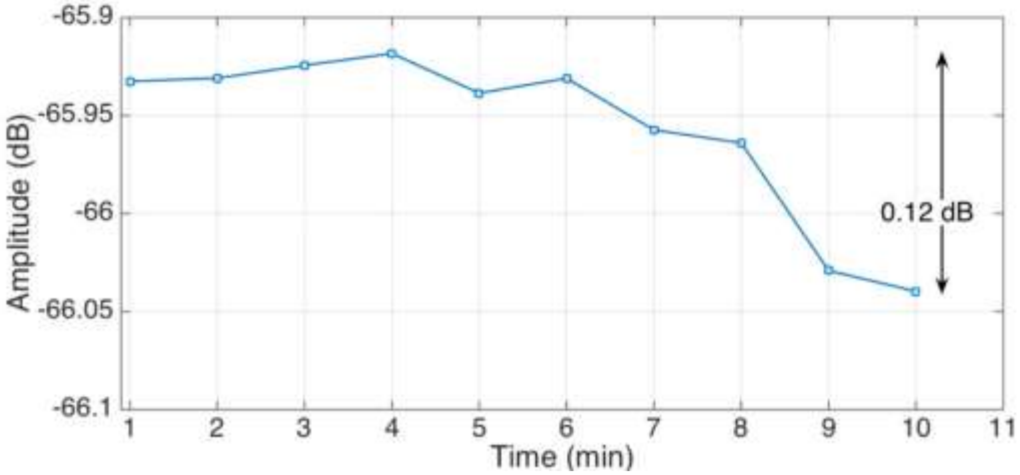

Figure S4. Amplitude change as a function of time during CD44 protein detection by functionalized fiber optic tip. The spectral response measured every 1 minute at 0.8 nM conc. of the protein (middle concentration) for 10 minutes.

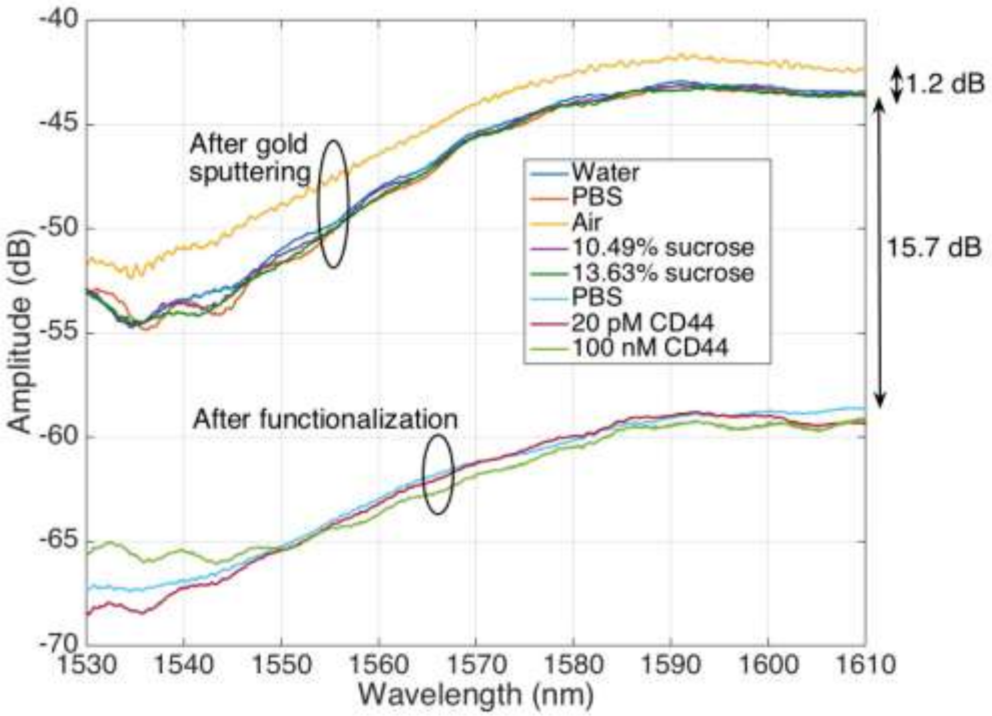

Figure S5. Evolution of the spectral amplitude of spherical fiber optic tip (CD44 protein detecting biosensor) after gold sputtering and after full functionalization. Spectra of the sensors in the range from 1530 to 1610 nm in different media are shown including the lowest/highest sucrose concentrations used during calibration; and two concentrations of CD44 protein.

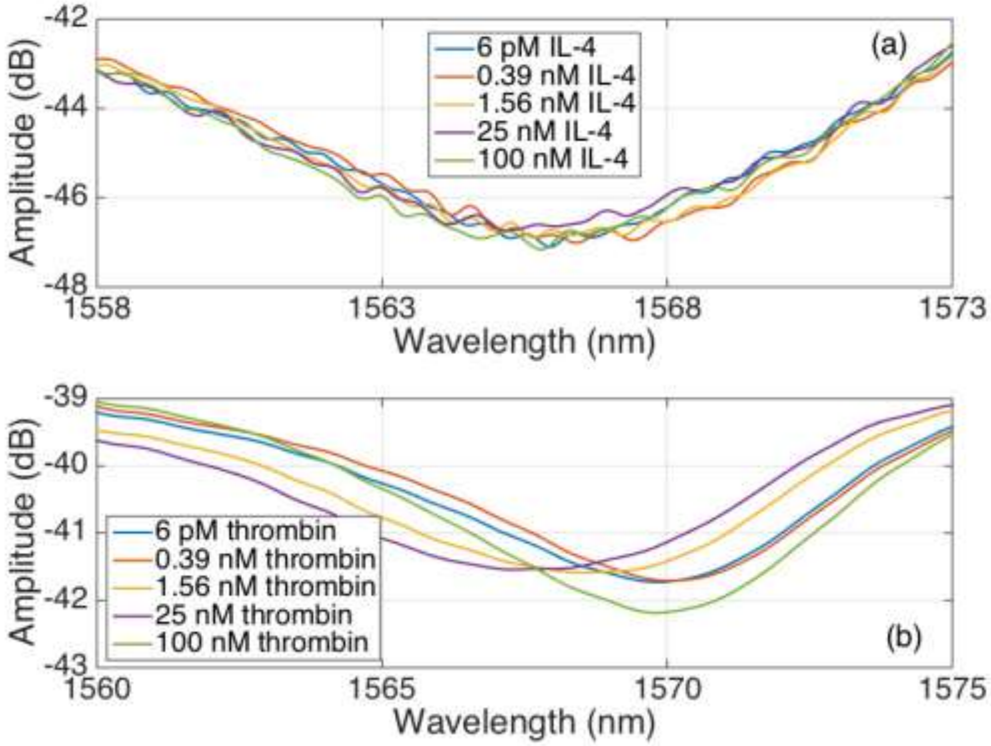

Figure S6.

Figure S6. Detection of control proteins by spherical fiber optic tip biosensor functionalized with CD44 antibodies; Spectral change occurring during measurement of different concentrations of (a) IL-4 and (b) thrombin by the biosensor is shown; Spectral response in the range shown (between 1558-1573 nm and 1560-1575 nm respectively) were integrated to compare the signal with those of the target protein (**Figure 5**).
